# High-Speed imaging reveals opposing effects of chronic stress and antidepressants on neuronal activity propagation through the hippocampal trisynaptic circuit

**DOI:** 10.3389/fncir.2015.00070

**Published:** 2015-11-06

**Authors:** Jens Stepan, Florian Hladky, Andrés Uribe, Florian Holsboer, Mathias V. Schmidt, Matthias Eder

**Affiliations:** ^1^Max Planck Institute of PsychiatryMunich, Germany; ^2^Department Stress Neurobiology and Neurogenetics, Max Planck Institute of PsychiatryMunich, Germany; ^3^Scientific Core Unit “Electrophysiology and Neuronal Network Dynamics”, Max Planck Institute of PsychiatryMunich, Germany; ^4^Clinical Department, Max Planck Institute of PsychiatryMunich, Germany; ^5^Research Group “Stress Resilience”, Max Planck Institute of PsychiatryMunich, Germany; ^6^HMNC GmbHMunich, Germany

**Keywords:** hippocampus, trisynaptic circuit, activity propagation, stress, antidepressants, ketamine, voltage-sensitive dye, imaging

## Abstract

Antidepressants (ADs) are used as first-line treatment for most stress-related psychiatric disorders. The alterations in brain circuit dynamics that can arise from stress exposure and underlie therapeutic actions of ADs remain, however, poorly understood. Here, enabled by a recently developed voltage-sensitive dye imaging (VSDI) assay in mouse brain slices, we examined the impact of chronic stress and concentration-dependent effects of eight clinically used ADs (belonging to different chemical/functional classes) on evoked neuronal activity propagations through the hippocampal trisynaptic circuitry (HTC: perforant path → dentate gyrus (DG) → area CA3 → area CA1). Exposure of mice to chronic social defeat stress led to markedly weakened activity propagations (“HTC-Waves”). In contrast, at concentrations in the low micromolar range, all ADs, which were bath applied to slices, caused an amplification of HTC-Waves in CA regions (invariably in area CA1). The fast-acting “antidepressant” ketamine, the mood stabilizer lithium, and brain-derived neurotrophic factor (BDNF) exerted comparable enhancing effects, whereas the antipsychotic haloperidol and the anxiolytic diazepam attenuated HTC-Waves. Collectively, we provide direct experimental evidence that chronic stress can depress neuronal signal flow through the HTC and demonstrate shared opposing effects of ADs. Thus, our study points to a circuit-level mechanism of ADs to counteract stress-induced impairment of hippocampal network function. However, the observed effects of ADs are impossible to depend on enhanced neurogenesis.

## Introduction

Stress is a major environmental risk factor for the development of several psychiatric diseases, including depression and anxiety disorders (de Kloet et al., 2005; Krishnan and Nestler, 2008; Popoli et al., 2012). To date, the first-line treatment for most stress-related psychiatric disorders is administration of antidepressants (ADs; Benkert and Hippius, 2012). While intensive research has provided fundamental insights into stress-induced brain abnormalities and mechanisms of AD action at the molecular, cellular, and synaptic level (Castrén, 2005; de Kloet et al., 2005; Krishnan and Nestler, 2008; Popoli et al., 2012; Hill et al., 2015), the alterations in millisecond-scale neuronal circuit dynamics that can arise from stress exposure and underlie therapeutic actions of ADs are largely unknown. Investigations on this topic appear crucial, since many symptoms of psychiatric disorders and their pharmacological alleviation are increasingly thought to be best reflected by changes in spatio-temporal patterns of electrical neuronal activity (Castrén, 2005; Airan et al., 2007; Karayiorgou et al., 2012; Monteggia et al., 2014).

**Figure 1:**
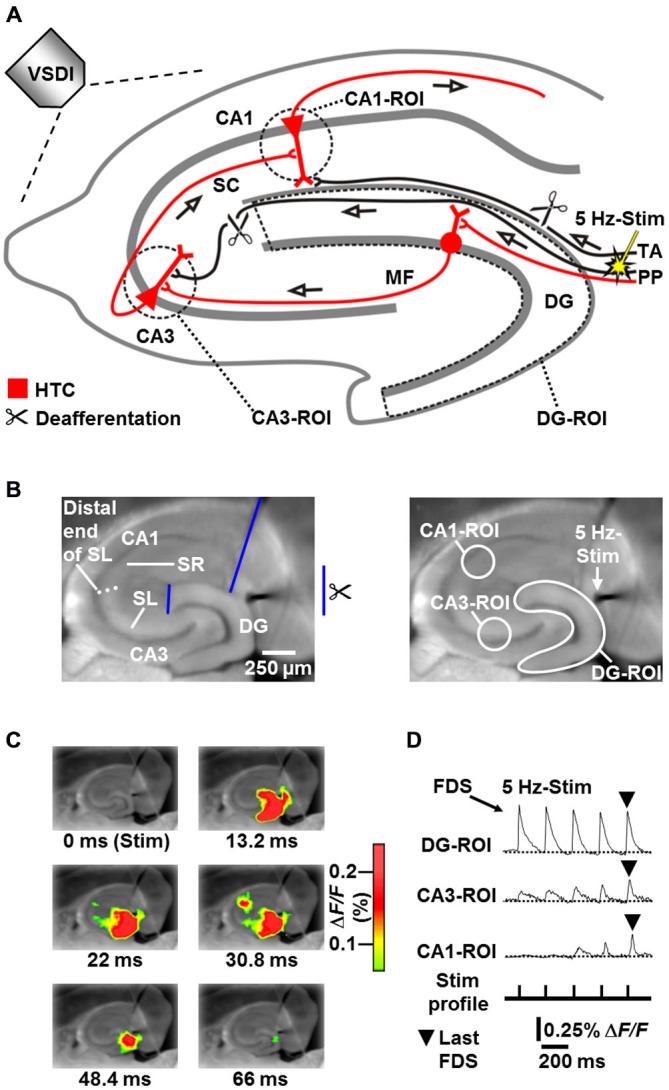
**Principle of the HTC-Wave Voltage-Sensitive Dye Imaging (VSDI) assay in mouse hippocampal slices. (A,B)** Experimental arrangement used to obtain the data shown in **(C)** and** (D)** (modified from Stepan et al., 2012). MF, mossy fiber; PP, perforant path; ROI, region of interest; SC, Schaffer collateral; SL, stratum lucidum; SR, stratum radiatum; Stim, extracellular electrical stimulation; TA, temporoammonic pathway. **(C)** VSDI filmstrip of a submaximal HTC-Wave evoked by theta-frequency (5 Hz) electrical stimulation of the perforant path (stimulus 5). Only selected imaging frames are shown (sampling time was 2.2 ms). Warmer colors represent higher values of the fractional change in fluorescence (Δ*F/F*) of the voltage-sensitive dye and, thus, stronger neuronal activity. For the appearance of maximal (saturated) HTC-Waves see Stepan et al. (2012). **(D)** Depiction of ROI-extracted fast, depolarization-mediated imaging signals (FDSs), which reflect neuronal action potentials and glutamatergic excitatory postsynaptic potentials (EPSPs). Note the typically increasing CA3 signals and the delayed occurrence of CA1-FDSs, which predominantly derive from frequency facilitation of neurotransmission at mossy fiber synapses onto CA3 pyramidal neurons (cf., supplementary movie in Stepan et al., 2012).

A brain structure that is highly sensitive to stress hormones, involved in the regulation of stress responses, and most likely a therapeutically relevant target of ADs is the hippocampus (Andersen et al., 2007; Maggio and Segal, 2007; von Wolff et al., 2011; Méndez et al., 2012; Hill et al., 2015). Stress can severely affect the structural integrity of the hippocampal network and it is widely accepted that at least a part of the resultant neurophysiological changes contribute to the symptomatology of depression and other stress-related psychiatric diseases (de Kloet et al., 2005; Popoli et al., 2012). A major function of the hippocampus is to process sensory information, to store it if appropriate, and to transmit it to downstream brain structures. Transfer of sensory information to the hippocampus prominently takes place via perforant path fibers (originating in entorhinal cortex layer II) that synapse on neurons of the dentate gyrus (DG). The DG represents the first relay station of the glutamatergic trisynaptic circuitry of the hippocampus (in the following abbreviated HTC: perforant path → DG → area CA3 → CA1 output subfield, Figure 1A; Andersen et al., 2007).

We recently developed a voltage-sensitive dye imaging (VSDI) assay in mouse brain slices, allowing real-time monitoring and, thus, the investigation of neuronal activity propagations through the entire HTC network (“HTC-Waves”; Stepan et al., 2012, 2015). In this assay (Figure 1), HTC-Waves are triggered by theta-frequency stimulation of perforant path fibers, mimicking synchronous theta-rhythmical spiking of entorhinal layer II neurons, which exhibit such activity during physiologically highly relevant theta oscillations in the entorhinal-hippocampal system (Dickson et al., 2000; Mizuseki et al., 2009; Quilichini et al., 2010; Burgalossi et al., 2011). Here, by use of the HTC-Wave assay, we are the first to examine the impact of chronic stress and concentration-dependent effects of ADs on polysynaptic activity propagations through a major input-output network of the hippocampus.

VSDI in brain slices has been already previously employed to investigate stress and AD effects on hippocampal circuit dynamics (Airan et al., 2007). In this work, rats were exposed to chronic mild stress or treated with fluoxetine or imipramine for several days. The authors found that a measure of local activity spread within the DG was decreased in slices from stressed animals and observed an opposing effect in area CA1. Antipodal neurophysiological changes were detected in slices from AD-treated animals. In contrast to the present study, neuronal activity in the DG and area CA1 was evoked independently from each other by local electrical stimulation, thus excluding measurements of activity propagation through the whole HTC network and potential effects on the highly stress-sensitive CA3 circuitry.

To summarize our main results, we found that exposure of mice to chronic social defeat stress leads to markedly weakened HTC-Waves, while all eight ADs tested (belonging to different chemical/functional classes) amplify the activity propagations in CA regions at concentrations in the low micromolar range.

## Materials and Methods

### Animals

For all experiments, adult male C57BL/6N mice (8 to 12 weeks old, obtained from the Max Planck Institute’s breeding colony) and adult male CD-1 mice (12 to 24 weeks old, purchased from Charles River) were used. All experimental procedures were approved by the committee for the Care and Use of Laboratory animals of the Government of Upper Bavaria, Germany.

### Chronic Social Defeat Stress

The stress paradigm lasted 14–19 days and was conducted as we described previously (Wagner et al., 2012). Briefly, the experimental C57BL/6N mice were introduced into the home cage of a dominant resident CD-1 mouse and defeated shortly after. When the defeat was achieved, the animals were separated by a wire mesh, preventing physical but allowing sensory contact for 24 h. Each day, animals of the stress group were defeated by another unfamiliar, dominant resident mouse, excluding a repeated encounter throughout the experiment. The daily defeat was performed between 11:00 am and 4:00 pm. Varying starting times reduced the predictability of the stressor and, therefore, minimized a potential habituation effect. Control mice were housed in their home cages during the course of the experiment. Both stressed and control animals were handled daily during the stress procedure. At the day of VSDI experimentation, animals of the stress group were not anymore defeated. VSDI measurements were done between day 15 and 20 after the start of the stress paradigm.

### Preparation and Staining of Brain Slices

C57BL/6N mice were anesthetized with isoflurane and decapitated. All following steps were done in ice-cold sucrose-based saline saturated with carbogen gas (95% O_2_/5% CO_2_). This saline (pH 7.4) consisted of (in mM): 87 NaCl, 2.5 KCl, 25 NaHCO_3_, 1.25 NaH_2_PO_4_, 0.5 CaCl_2_, 7 MgCl_2_, 25 glucose, and 75 sucrose. The hemispheres were prepared for the slicing procedure by a special transversal cut, which is sometimes called “magic cut” (Bischofberger et al., 2006). We cut the hemispheres at angles optimized to conserve the intrahippocampal axonal projections along the DG-CA axis as best as possible. Subsequently, 350 μm-thick horizontal slices were cut using a vibratome (HM650V, Thermo Scientific). Slices were incubated in carbogenated sucrose-based saline for 30 min at 34°C. Subsequent staining with the voltage-sensitive dye Di-4-ANEPPS (dissolved in DMSO to a 20.8 mM stock solution) was carried out at room temperature (23–25°C). Slices were kept for 15 min in carbogenated physiological saline containing Di-4-ANEPPS (7.5 μg/ml, <0.1% DMSO). The physiological saline (pH 7.4) consisted of (in mM): 125 NaCl, 2.5 KCl, 25 NaHCO_3_, 1.25 NaH_2_PO_4_, 2 CaCl_2_, 1 MgCl_2_, and 25 glucose. Afterwards, slices were stored at room temperature for at least 30 min in Di-4-ANEPPS-free carbogenated physiological saline containing 0.6 μM bicuculline methiodide (BIM, for rationale see “Discussion” Section).

### VSDI

VSDI and data analysis were performed using the MiCAM02 hard and software package (BrainVision). The tandem-lens fluorescence microscope was equipped with the MiCAM02-HR camera and the 2× and 1× lens at the objective and condensing side, respectively (for further technical details see http://www.scimedia.com). Acquisition settings were as follows: 88 × 60 pixels frame size, 36.4 × 40.0 μm pixel size, and 2.2 ms sampling time.

### Processing and Quantification of VSDI Data

From VSDI signals, the fractional change in fluorescence (Δ*F/F*) was calculated and Δ*F/F* values were spatially and temporally smoothed using a 3 × 3 × 3 average filter. VSDI signals presented in images were smoothed with a 5 × 5 × 3 average filter. To attenuate slow signal components produced from bleaching of the dye and slight summation of 5 Hz neuronal responses (see next paragraph), we afterwards applied a weak high-pass filter of the MiCAM02 software (τ = 220 ms) to the imaging data. Pixelation of images was reduced by the interpolation function of the MiCAM02 software.

For analysis of neuronal population activity in hippocampal subregions, standardized regions of interest (ROIs) were manually set according to anatomical landmarks. The circular CA3-ROI (*r* = 4 pixels) was positioned into the CA3 region near the DG, but not overlapping with it. The circular CA1-ROI (*r* = 4 pixels) was placed into the CA1 subfield with a distance of approximately 200 μm from the visually identified distal end of the stratum lucidum. Both ROIs spanned the stratum oriens, stratum pyramidale, and stratum lucidum/radiatum. The DG-ROI, which enclosed the fascia dentata (Figures 1A,B), was created by the polygon-drawing function of the MiCAM02 software. The average of smoothed Δ*F/F* values within a particular ROI served as final measure of neuronal population activity.

### Brain Slice Experiments

All slice experiments were carried out at room temperature and slices were continuously superfused with BIM (0.6 μM)-containing carbogenated physiological saline (4–5 ml/min flow rate). BIM never led to epileptiform activity in the hippocampal subfields under study (for the appearance of such activity see Stepan et al., 2012). HTC-Waves were evoked by square pulse electrical stimuli (200 μs pulse width) delivered at 5 Hz via a monopolar tungsten electrode (50 μm tip diameter, ~0.5 MΩ nominal impedance) to the visually identified perforant path near its entry zone to the DG (Figures 1A,B). In all slices, perforant path fibers which directly innervate CA3 pyramidal cells were cut at the point where they exit the DG. Temporoammonic projections were likewise functionally inactivated (Figures 1A,B). For the pharmacological experiments, the intensity of perforant path stimulation (15–35 V) was adjusted in a manner to produce fast, depolarization-mediated imaging signals (FDSs) in the DG with amplitudes of 50–80% of the highest attainable value. These FDSs range within the linear upturn of the respective input-output curve (Stepan et al., 2012). To obtain submaximal HTC-Waves comprising CA1-FDSs with amplitudes of 0.1–0.3% Δ*F/F*, 4–7 consecutive stimulation pulses were delivered per recording sequence to the perforant path (Figures 1C,D). The number of consecutive stimulation pulses was held constant within an experiment. Acquisitions were made every 2 min and the amplitude values of the last DG-, CA3-, and CA1-FDS within a recording sequence (Figure 1D) were determined by means of the MiCAM02 software. As final measure of DG, CA3, and CA1 activity, we calculated mean DG-, CA3-, and CA1-FDS amplitude values over three consecutive acquisitions. Drugs were bath applied to slices if baseline recording was stable over 20 min (Figure 3A). For each pharmacological condition, a maximum number of two slices per animal was used.

**Figure 2:**
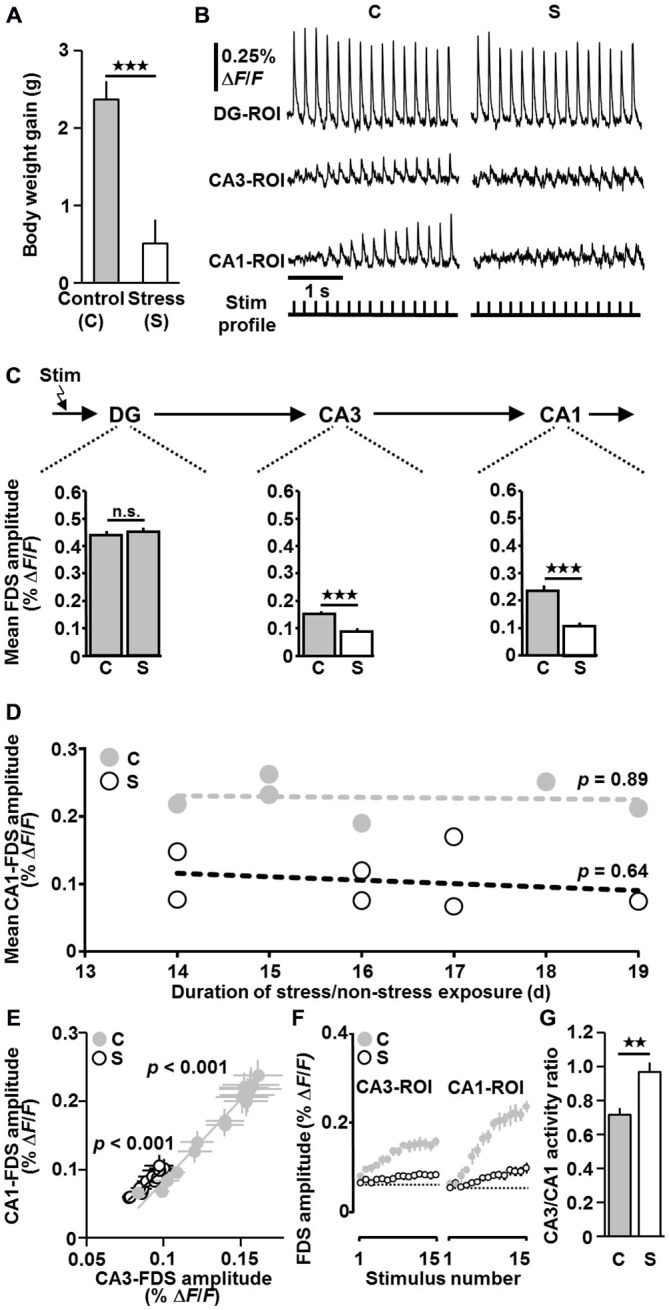
**Exposure of mice to chronic social defeat stress causes markedly weakened HTC-Waves. (A)** Data from body weight measurements (body weight gain during stress/non-stress exposure; stress (*n* = 7) vs. control (*n* = 6): *t*_(11)_ = −4.6, *p* < 0.001; two-tailed unpaired *t*-test). **(B)** Representative VSDI recording traces for control and stress conditions. For both recording sequences, the intensity of perforant path stimulation (15 pulses at 5 Hz) was adjusted such that the first DG-FDS had an amplitude of 0.45% Δ*F/F*. **(C)** Quantification of VSDI measurements (*n* = 6 animals/18 slices for the control group and *n* = 7 animals/19 slices for the stress group; VSDI measures obtained from 2–3 slices per animal were averaged, leading to *n* values of 6 and 7 for the control and stress group, respectively; stress vs. control: CA3: *t*_(11)_ = −5.0, *p* < 0.001; CA1: *t*_(11)_ = −5.7, *p* < 0.001; two-tailed unpaired *t*-tests). For a detailed description of how the measure “Mean FDS amplitude” was calculated see “Materials and Methods”. **(D)** Relationships between mean CA1-FDS amplitude and duration of stress/non-stress exposure (*p* values were determined by linear regression analyses). **(E)** In slices from both control and stressed mice, the amplitude of CA1-FDSs linearly increased with the amplitude of CA3-FDSs. Data were obtained by averaging the CA1- and CA3-FDS amplitude values for electrical stimulus 1, 2, 3, …, 15 over the 18/19 experiments performed in control/stressed mice, in which the amplitude of the first DG-FDS was adjusted to ~0.45% Δ*F/F*. **(F)** The typical increase in CA3- and CA1-FDSs during 5 Hz perforant path stimulation is much weaker in slices from stressed animals. **(G)** CA3/CA1 activity ratios for the experiments conducted in control/stressed mice, in which the amplitude of the first DG-FDS was adjusted to 0.45% Δ*F/F* (stress vs. control: *t*_(11)_ = 3.5, *p* = 0.005; two-tailed unpaired *t*-test). **p < 0.01, ***p < 0.001; n.s., not statistically significant.

For the experiments in slices from stressed/non-stressed mice, we used a HTC-Wave assay, which was refined for group comparisons. We established an experimental procedure, by which inter-slice variability in HTC dynamics could be well handled. For each animal, this procedure was applied to 2–3 slices. In particular, we delivered three trains of 15 stimulation pulses (5 Hz stimulation, 3 min inter-train interval) to the perforant path. The stimulation intensity was adjusted such that the first DG-FDS within recording sequence one, two, and three had an amplitude of approximately 0.35, 0.45, and 0.55% Δ*F/F*, respectively (Figure 2B). The resultant HTC-Waves comprised CA3- and CA1-FDSs, which linearly increased with increasing stimulation intensity (data not shown). For quantification, we used the amplitude value of the first DG-FDS, the mean amplitude value of the last three CA3-FDSs, and the mean amplitude value of the last three CA1-FDSs within a recording sequence. These values were averaged over the three recording sequences. The resultant mean values in turn were averaged over the 2–3 experiments conducted for each animal, yielding the measure “Mean FDS amplitude” (Figure 2C). Healthiness of “stress” slices was comparable to that of “control” slices, since the ranges of stimulation intensities needed for evocation of the defined DG-FDSs were nearly identical (6–32 V for “stress” slices and 7–36 V for “control” slices).

### Chemicals

Amitriptyline hydrochloride, BIM, citalopram hydrobromide, clomipramine hydrochloride, Di-4-ANEPPS, diazepam (dissolved in DMSO to a 100 mM stock solution, ≤0.001% final DMSO), DMSO, fluoxetine hydrochloride, fluvoxamine maleate, haloperidol (dissolved in DMSO to a 100 mM stock solution, ≤0.02% final DMSO), S-(+)-ketamine hydrochloride, LiCl, tranylcypromine hydrochloride, venlafaxine hydrochloride, and all salts for the saline solutions were purchased from Sigma-Aldrich. ANA-12 and tianeptine sodium salt were from Tocris, brain-derived neurotrophic factor (BDNF) from Biomol, and isoflurane from Abbott.

### Statistics

Statistical analyses were run in SigmaStat (Systat Software), with significance declared at *p* < 0.05 (*p < 0.05, **p < 0.01, ***p < 0.001; n.s., not statistically significant). Data are given as mean ± SEM. Statistical tests are described in the respective paragraphs of the results section and/or figure legends. In the pharmacological experiments where ANOVAs were employed for statistical evaluation, the one-sample *t*-test was used to determine the concentration(s) at which a statistically significant effect took place, given that the ANOVA passed (*p* < 0.05).

## Results

### Exposure of Mice to Chronic Social Defeat Stress Causes Markedly Weakened HTC-Waves

We examined the impact of chronic social defeat stress (14–19 days) on HTC-Waves. To assure “pure” activity propagations through the HTC network, perforant path fibers which directly innervate CA3 pyramidal cells were cut at the point where they exit the DG. Temporoammonic projections were likewise functionally inactivated (Figures 1A,B (scissors)). As a real-time measure of neuronal population activity in the DG and CA areas, we employed the amplitude of fast, depolarization-mediated VSDI signals (FDSs, Figure 1D), which reflect action potentials and glutamatergic excitatory postsynaptic potentials (EPSPs; Airan et al., 2007; von Wolff et al., 2011; Stepan et al., 2012). For more detailed information about the characteristics and physiological relevance of HTC-Waves see “Discussion” Section and Stepan et al. (2012, 2015). The stress paradigm used reliably induces changes in metabolic and endocrine parameters, which typically occur with chronic stress exposure. Furthermore, it causes an increased stress reactivity and an impairment of hippocampus-dependent cognitive abilities (Wang et al., 2011; Wagner et al., 2012). Body weight measurements confirmed that the mice under investigation were profoundly stressed (Figure 2A; Wang et al., 2011). As evident from a pronounced decrease of CA3- and CA1-FDSs, the stress procedure led to considerably weakened HTC-Waves when compared to control conditions (Figures 2B,C). Linear regression analyses showed that there was no relationship between this effect and the duration of stress exposure (Figure 2D).

Further analyses revealed that in slices from both control and stressed mice the amplitude of CA1-FDSs linearly increased with the amplitude of CA3-FDSs (Figure 2E). However, the typical initial increase in CA3- and CA1-FDSs during 5 Hz perforant path stimulation, which predominantly derives from frequency facilitation of neurotransmission at mossy fiber synapses onto CA3 pyramidal neurons (Stepan et al., 2012), was much weaker in slices from stressed animals (Figure 2F). We also calculated CA3/CA1 activity ratios for “control” and “stress” slices. These ratios were determined by dividing the mean amplitude value of the last three CA3-FDSs by the mean amplitude value of the last three CA1-FDSs within a recording sequence and by averaging the resultant quotients over the 2–3 experiments conducted for each animal. The CA3/CA1 activity ratio was higher in the stress group (Figure 2G). These findings strongly suggest that the stress effect on HTC-Waves originated from impaired neurotransmission in both area CA3 and area CA1.

### ADs Exert Enhancing Effects on HTC-Waves

**Figure 3:**
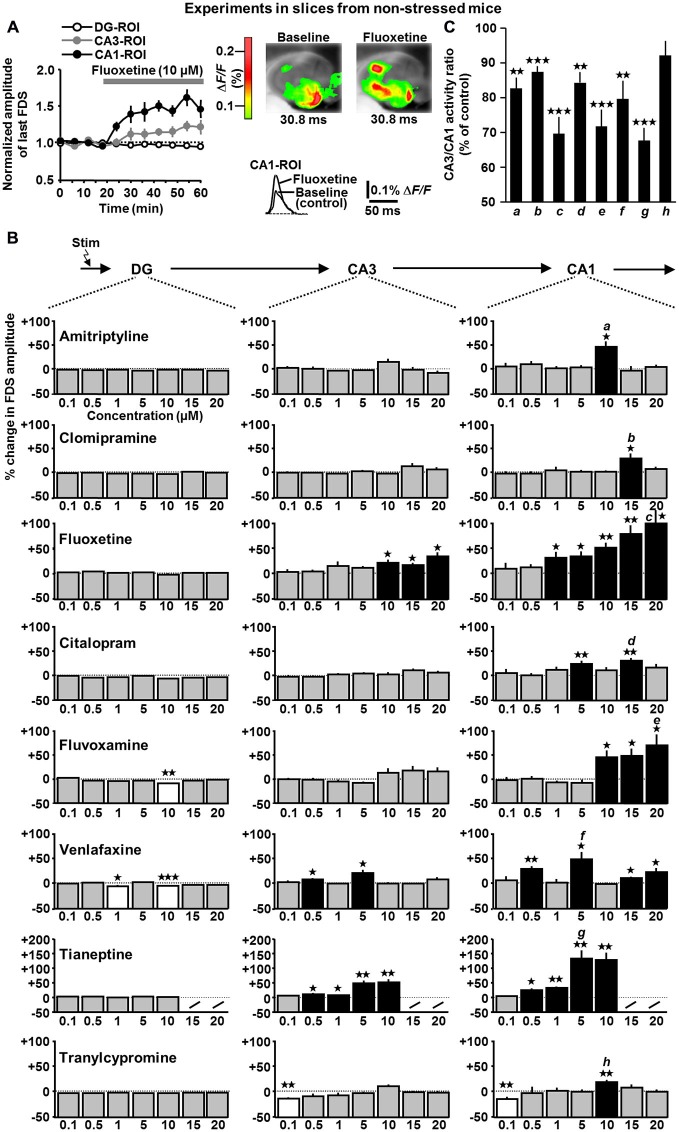
**Time course of fluoxetine effects (A) and concentration-dependent impact of amitriptyline, clomipramine, fluoxetine, citalopram, fluvoxamine, venlafaxine, tianeptine, and tranylcypromine (B) on HTC-Waves in slices from non-stressed mice. (A)** As done with all other drugs probed, fluoxetine (10 μM) was bath applied to slices (*n* = 7) if baseline recording of HTC-Waves was stable over 20 min. Subsequent recording time was always 40 min. Data were normalized to mean baseline FDS amplitude values. **(B)** For statistical analysis, one-way ANOVAs were applied to the DG-, CA3-, and CA1-FDS data sets (% change in FDS amplitude, minutes 42–60 vs. minutes 0–18 of VSDI recording **(A)**) covering the whole range of concentrations probed. Due to its very strong effects at 5 and 10 μM, tianeptine was no longer tested at 15 and 20 μM. DG: fluvoxamine: *F*_(6,50)_ = 4.2, *p* = 0.002; 10 μM, *t*_(7)_ = −5.3, *p* = 0.001; venlafaxine: *F*_(6,52)_ = 2.7, *p* = 0.021; 1 μM, *t*_(8)_ = −3.1, *p* = 0.014; 10 μM, *t*_(8)_ = −9.5, *p* < 0.001; CA3: fluoxetine: *F*_(6,47)_ = 2.5, *p* = 0.033; 10 μM, *t*_(6)_ = 3.5, *p* = 0.013; 15 μM, *t*_(6)_ = 3.4, *p* = 0.013; 20 μM, *t*_(7)_ = 3.4, *p* = 0.011; venlafaxine: *F*_(6,52)_ = 4.2, *p* = 0.002; 0.5 μM, *t*_(9)_ = 2.6, *p* = 0.027; 5 μM, *t*_(7)_ = 3.0, *p* = 0.019; tianeptine: *F*_(4,30)_ = 8.8, *p* < 0.001; 0.5 μM, *t*_(6)_ = 2.8, *p* = 0.031; 1 μM, *t*_(6)_ = 2.6, *p* = 0.043; 5 μM, *t*_(6)_ = 4.0, *p* = 0.007; 10 μM, *t*_(6)_ = 4.4, *p* = 0.005; tranylcypromine: *F*_(6,56)_ = 3.7, *p* = 0.004; 0.1 μM, *t*_(7)_ = −4.6, *p* = 0.002; CA1: amitriptyline: *F*_(6,46)_ = 4.2, *p* = 0.002; 10 μM, *t*_(6)_ = 3.3, *p* = 0.016; clomipramine: *F*_(6,59)_ = 2.8, *p* = 0.02; 15 μM, *t*_(8)_ = 2.8, *p* = 0.023; fluoxetine: *F*_(6,47)_ = 4.6, *p* < 0.001; 1 μM, *t*_(7)_ = 2.4, *p* = 0.047; 5 μM, *t*_(7)_ = 3.3, *p* = 0.013; 10 μM, *t*_(6)_ = 5.2, *p* = 0.002; 15 μM, *t*_(6)_ = 4.2, *p* = 0.006; 20 μM, *t*_(7)_ = 3.4, *p* = 0.01; citalopram: *F*_(6,51)_ = 2.3, *p* = 0.049; 5 μM, *t*_(9)_ = 3.7, *p* = 0.005; 15 μM, *t*_(7)_ = 4.9, *p* = 0.002; fluvoxamine: *F*_(6,50)_ = 5.9, *p* < 0.001; 10 μM, *t*_(7)_ = 2.9, *p* = 0.023; 15 μM, *t*_(7)_ = 3.1, *p* = 0.017; 20 μM, *t*_(8)_ = 3.2, *p* = 0.016; venlafaxine: *F*_(6,52)_ = 4.8, *p* < 0.001; 0.5 μM, *t*_(9)_ = 4.6, *p* = 0.001; 5 μM, *t*_(7)_ = 3.0, *p* = 0.019; 15 μM, *t*_(6)_ = 2.9, *p* = 0.014; 20 μM, *t*_(7)_ = 3.4, *p* = 0.022; tianeptine: *F*_(4,30)_ = 11.2, *p* < 0.001; 0.5 μM, *t*_(6)_ = 3.6, *p* = 0.012; 1 μM, *t*_(6)_ = 5.8, *p* = 0.001; 5 μM, *t*_(6)_ = 4.6, *p* = 0.004; 10 μM, *t*_(6)_ = 4.6, *p* = 0.004; tranylcypromine: *H*_(6)_ = 18.6, *p* = 0.005 (Kruskal-Wallis ANOVA on ranks); 0.1 μM, *t*_(7)_ = −3.6, *p* = 0.008; 10 μM, *t*_(7)_ = 4.4, *p* = 0.003. Number of experiments (slices) for 0.1, 0.5, 1, 5, 10, 15, and 20 μM: amitriptyline: *n* = 8, 8, 8, 7, 7, 7, and 8; clomipramine: *n* = 9, 12, 10, 10, 8, 9, and 8; fluoxetine: *n* = 8, 8, 8, 8, 7, 7, and 8; citalopram: *n* = 8, 9, 8, 10, 8, 8, and 7; fluvoxamine: *n* = 8, 8, 8, 8, 8, 8, and 9; venlafaxine: *n* = 8, 10, 9, 8, 9, 7, and 8; tianeptine: *n* = 7, 7, 7, 7, and 7; tranylcypromine: *n* = 8, 10, 8, 8, 8, 13, and 8. For each pharmacological condition, a maximum number of two slices per animal was used. **(C)** Relative change in CA3/CA1 activity ratios in the presence of 10 μM amitriptyline (*a*), 15 μM clomipramine (*b*), 20 μM fluoxetine (*c*), 15 μM citalopram (*d*), 20 μM fluvoxamine (*e*), 5 μM venlafaxine (*f*), 5 μM tianeptine (*g*), and 10 μM tranylcypromine (*h*; amitriptyline: *t*_(6)_ = −5.4, *p* = 0.002; clomipramine: *t*_(8)_ = −7.8, *p* < 0.001; fluoxetine: *t*_(7)_ = −6.1, *p* < 0.001; citalopram: *t*_(7)_ = −5.1, *p* = 0.001; fluvoxamine: *t*_(8)_ = −5.8, *p* < 0.001; venlafaxine: *t*_(7)_ = −3.9, *p* = 0.008; tianeptine: *t*_(6)_ = −8.6, *p* < 0.001; one-sample *t*-tests). *p < 0.05, **p < 0.01, ***p < 0.001.

In another set of experiments, we investigated potential effects of eight clinically used ADs on HTC-Waves. The ADs were the tricyclics amitriptyline and clomipramine, the selective serotonin reuptake inhibitors fluoxetine, citalopram, and fluvoxamine, the selective serotonin/norepinephrine reuptake inhibitor venlafaxine, the selective serotonin reuptake enhancer tianeptine, and the monoamine oxidase A and B inhibitor tranylcypromine (McEwen et al., 2010; Benkert and Hippius, 2012). The ADs were tested at 0.1, 0.5, 1, 5, 10, and mostly also at 15 and 20 μM. As illustrated for fluoxetine (10 μM), which significantly increased CA3- and CA1-FDSs, drugs were bath applied to slices if baseline recording of HTC-Waves was stable over 20 min (Figure 3A).

With the exception of fluvoxamine (10 μM) and venlafaxine (1 and 10 μM), the ADs did not affect DG-FDSs (Figures 3A,B (left panels)). In contrast, at concentrations in the low micromolar range, all ADs led in the same experiments to an enhancement of CA1-FDSs (Figures 3A,B (black bars in right panels)). Interestingly, only the data obtained for fluoxetine, fluvoxamine, and tianeptine are reconcilable with a classical dose-response relationship. Fluoxetine (10, 15, and 20 μM), venlafaxine (0.5 and 5 μM), and tianeptine (0.5, 1, 5, and 10 μM) additionally increased CA3-FDSs (Figures 3A,B (black bars in middle panels)). Another finding was that tranylcypromine (0.1 μM) decreased CA3- and CA1-FDSs (Figure 3B). With the exception of tranylcypromine, all ADs reduced the CA3/CA1 activity ratio (Figure 3C).

Taken together, at concentrations in the low micromolar range (but not at 0.1 μM and mostly also not at 0.5 μM), all ADs caused an amplification of HTC-Waves in the CA1 output subfield of the hippocampus. At some of the effective doses, fluoxetine, venlafaxine, and tianeptine also provoked an enhancement of CA3 activity. A close inspection of the data depicted in Figure 3B suggests that the latter effect, although lacking statistical significance, likewise occurred with the other ADs.

### Lithium, BDNF, and Ketamine Exert Enhancing Effects on HTC-Waves

**Figure 4:**
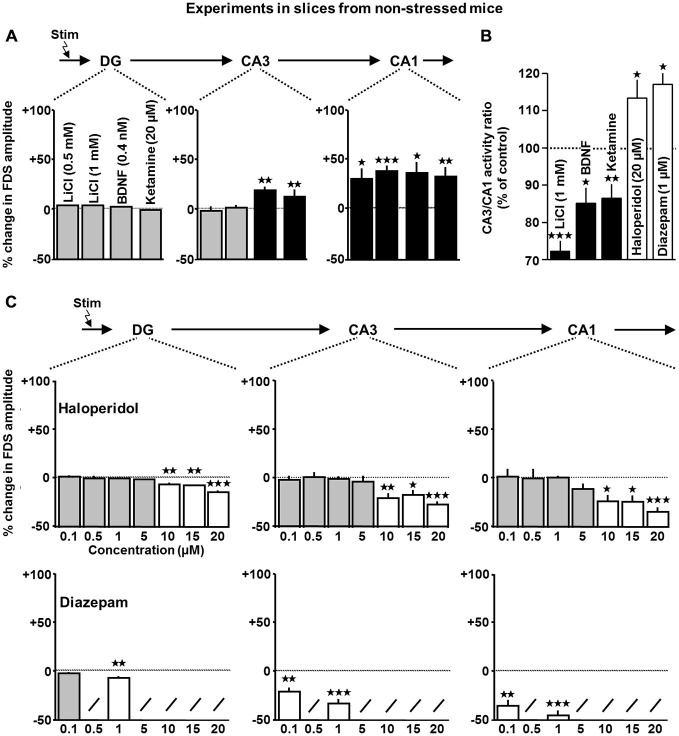
**Impact of lithium, BDNF, ketamine, haloperidol, and diazepam on HTC-Waves in slices from non-stressed mice. (A)** For statistical analysis, one-sample *t*-tests were used (lithium (*n* = 7 slices for 0.5 and 1 mM): CA1: 0.5 mM, *t*_(6)_ = 2.5, *p* = 0.045; 1 mM, *t*_(6)_ = 7.5, *p* < 0.001; BDNF (*n* = 8 slices): CA3: *t*_(7)_ = 3.6, *p* = 0.008; CA1: *t*_(7)_ = 3.4, *p* = 0.011; ketamine (*n* = 8 slices): CA3: *t*_(7)_ = 4.9, *p* = 0.002; CA1: *t*_(7)_ = 4.2, *p* = 0.004). **(B)** Relative change in CA3/CA1 activity ratios in the presence of 1 mM lithium (*a*), 0.4 nM BDNF (*b*), 20 μM ketamine (c), 20 μM haloperidol (*d*), and 1 μM diazepam (*e*; lithium: *t*_(6)_ = −9.7, *p* < 0.001; BDNF: *t*_(7)_ = −3.5, *p* = 0.01; ketamine: *t*_(7)_ = −3.6, *p* = 0.009; haloperidol: *t*_(5)_ = 2.7, *p* = 0.035; diazepam: *t*_(5)_ = 3.3, *p* = 0.029; one-sample *t*-tests). **(C)** Statistical evaluation of haloperidol effects was performed as done for ADs. For diazepam effects, the one-sample *t*-test was used (haloperidol: DG: *F*_(6,43)_ = 17.8, *p* < 0.001; 10 μM, *t*_(7)_ = −4.3, *p* = 0.003; 15 μM, *t*_(5)_ = −4.8, *p* = 0.005; 20 μM, *t*_(5)_ = −10.1, *p* < 0.001; CA3: *F*_(6,43)_ = 4.3, *p* = 0.002; 10 μM, *t*_(7)_ = −3.7, *p* = 0.007; 15 μM, *t*_(5)_ = −3.0, *p* = 0.028; 20 μM, *t*_(5)_ = −8.7, *p* < 0.001; CA1: *F*_(6,43)_ = 3.6, *p* = 0.005; 10 μM, *t*_(7)_ = −3.5, *p* = 0.01; 15 μM, *t*_(5)_ = −3.5, *p* = 0.017; 20 μM, *t*_(5)_ = −9.0, *p* < 0.001; number of experiments (slices) for 0.1, 0.5, 1, 5, 10, 15, and 20 μM: *n* = 8, 8, 6, 8, 8, 6, and 6; diazepam (*n* = 7 slices for 0.1 μM and *n* = 6 slices for 1 μM): DG: 1 μM, *t*_(5)_ = −4.1, *p* = 0.009; CA3: 0.1 μM, *t*_(6)_ = −4.9, *p* = 0.003; 1 μM, *t*_(5)_ = −7.8, *p* < 0.001; CA1: 0.1 μM, *t*_(6)_ = −5.7, *p* = 0.001; 1 μM, *t*_(5)_ = −8.4, *p* < 0.001). For each pharmacological condition, a maximum number of two slices per animal was used. *p < 0.05, **p < 0.01, ***p < 0.001.

We further tested whether other neuroactive factors, which do not belong to the group of classical ADs, but exhibit antidepressant-like actions, exert enhancing effects on HTC-Waves. We examined potential effects of the mood stabilizer lithium (Moore et al., 2002), the neurotrophin BDNF (Shirayama et al., 2002), and the fast-acting “antidepressant” ketamine (Autry et al., 2011) on the activity propagations. In fact, without changing DG-FDSs, lithium (0.5 and 1 mM; Moore et al., 2002), BDNF (0.4 nM; Kang and Schuman, 1995), and ketamine (20 μM; Autry et al., 2011) increased CA1-FDSs. BDNF and ketamine additionally caused an enhancement of CA3-FDSs (Figure 4A). As observed for most of the ADs, all three compounds reduced the CA3/CA1 activity ratio (Figure 4B).

### Haloperidol and Diazepam Weaken HTC-Waves

The findings described above led us to hypothesize that, in comparison to other psychiatric drugs (e.g., antipsychotics and anxiolytics), an enhancing influence on HTC-Waves might be a characteristic feature of ADs. On this account, we next performed experiments with the classical antipsychotic haloperidol and the widely used anxiolytic diazepam (Benkert and Hippius, 2012). In line with our hypothesis, haloperidol decreased DG-, CA3-, and CA1-FDSs in a concentration-dependent manner. Consistent with its potentiating effect on GABA_A_ receptor function and our previous demonstration that HTC-Waves are confined in their strength by GABAergic neurotransmission (Stepan et al., 2012), also diazepam (0.1 and 1 μM; Rupprecht et al., 2009) weakened the activity propagations (Figure 4C). In contrast to ADs, lithium, BDNF, and ketamine, haloperidol and diazepam increased the CA3/CA1 activity ratio (Figure 4B).

### Fluoxetine Exerts Enhancing Effects on HTC-Waves in Slices from Stressed Mice

**Figure 5:**
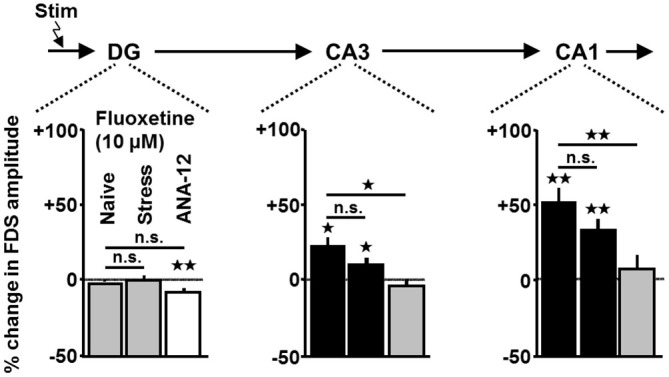
**Enhancing effects of fluoxetine on HTC-Waves occur in slices from chronically stressed mice and are prevented by the TrkB receptor antagonist ANA-12.** For group comparisons, two-tailed unpaired *t*-tests were used. For statistical analyses of fluoxetine effects in slices (*n* = 8) from stressed mice and ANA-12-treated slices (*n* = 11), one-sample *t*-tests were employed (stress: CA3: *t*_(7)_ = 2.4, *p* = 0.048; CA1: *t*_(7)_ = 4.7, *p* = 0.002; ANA-12: DG: *t*_(10)_ = −3.8, *p* = 0.003; for statistics of fluoxetine effects in naive slices see Figure 3; ANA-12 vs. naive: CA3: *t*_(16)_ = −2.8, *p* = 0.012; CA1: *t*_(16)_ = −3.4, *p* = 0.003). For each pharmacological condition, a maximum number of two slices per animal was used. *p < 0.05, **p < 0.01; n.s., not statistically significant.

Next, we addressed the question of whether ADs also exert enhancing effects on HTC-Waves in slices obtained from chronically stressed mice. Hereunto, we investigated the impact of fluoxetine (10 μM) on the activity propagations. We chose fluoxetine since it represents the classical AD, which showed the most pronounced effects on HTC-Waves (Figure 3B). Indeed, as observed in naive slices (for statistics see Figure 3), fluoxetine increased CA3- and CA1-FDSs in slices from stressed animals (Figure 5 (middle bars)).

### The TrkB Receptor Antagonist ANA-12 Prevents Fluoxetine Effects on HTC-Waves

There is substantial evidence that activation of the BDNF receptor TrkB is involved in the mechanisms of action of ADs (Castrén and Rantamäki, 2010). Our finding that BDNF, like ADs, increases FDSs in CA regions led us to test a possible contribution of TrkB activation to the AD effects on HTC-Waves. Hereunto, we preincubated slices for at least 1 h with the TrkB antagonist ANA-12 (10 μM; Longo and Massa, 2013). Afterwards, we again investigated the impact of fluoxetine (10 μM, for rationale see above) on HTC-Waves. As shown in Figure 5 (right bars), ANA-12 prevented the fluoxetine-induced amplification of CA1- and CA3-FDSs.

## Discussion

In the present study, we show that eight clinically used ADs (although belonging to different chemical/functional classes) cause an amplification of HTC-Waves in CA regions (invariably in the CA1 subfield and also partly in area CA3). However, these effects only occurred at concentrations in the low micromolar range, with the exception of venlafaxine and tianeptine, which additionally slightly boosted the activity propagations at 0.5 μM. Low micromolar brain concentrations of ADs are clinically relevant (Strauss et al., 1997; Bolo et al., 2000; Henry et al., 2005; Méndez et al., 2012), but considerably higher than those required for monoamine reuptake inhibition (IC_50_ values for serotonin reuptake inhibition by amitriptyline, clomipramine, fluoxetine, citalopram, and fluvoxamine (in nM): 39, 1.5, 6.8, 1.8, and 3.8, respectively (Hyttel, 1993)). In combination with our findings that also the serotonin reuptake enhancer tianeptine and BDNF increase CA3- and CA1-FDSs and that the TrkB receptor antagonist ANA-12 prevents the effects of fluoxetine (10 μM), it is thus possible that the AD effects observed do not depend on enhanced monoaminergic neurotransmission, but, at least in part, on increased TrkB signaling. Consistently, activation of TrkB is involved in AD actions and also lithium and ketamine, which likewise amplified HTC-Waves, enforce TrkB signaling (Castrén and Rantamäki, 2010; Autry et al., 2011).

Notably, only the data obtained for fluoxetine, fluvoxamine, and tianeptine are reconcilable with a classical dose-response relationship. The lack of such a relationship observed for the other five ADs and the fact that three compounds produced a boosting effect on HTC-Waves merely at one of the concentrations probed presumably rely on the partly intricate and heterogeneous pharmacological properties of ADs and the high structural and functional complexity of the HTC network (i.e., trisynaptic interconnections and appendant excitatory/inhibitory microcircuits; Andersen et al., 2007). This might also be the reason why fluvoxamine (10 μM), venlafaxine (1 and 10 μM), and tranylcypromine (0.1 μM) decreased DG- or CA3- and CA1-FDSs (Figure 3B). For instance, beside its enhancing action on monoaminergic neurotransmission, amitriptyline inhibits nicotinic acetylcholine receptors, use-dependently blocks voltage-gated sodium channels, and activates TrkB receptors with different potencies (Schofield et al., 1981; Huang et al., 2006; Jang et al., 2009). Similar (but not identical) pharmacological profiles have been described for some of the other ADs tested, whereas those of venlafaxine, tianeptine, and tranylcypromine are considerably different (Huang et al., 2006; Castrén and Rantamäki, 2010; McEwen et al., 2010; Benkert and Hippius, 2012). It is thus conceivable that a particular AD (at concentration A) exerts an enhancing effect on HTC-Waves, while, at a higher concentration B, this effect is less pronounced or absent (cf., Figure 3B (upper right panel)). Another AD in turn might show a varying effect pattern. Therefore, we deem it extremely challenging, if not impossible, to accurately predict AD actions on large/meso-scale neuronal network dynamics from known effects at the molecular/cellular level, given that these effects presumably have not been completely elucidated until now.

We yield evidence that, in comparison to antipsychotics and anxiolytics, enhancing effects on HTC-Waves are a characteristic feature of ADs and other neuroactive factors, which show antidepressant-like actions. This evidence is given by our findings that lithium, BDNF, and ketamine also cause an amplification of HTC-Waves, whereas haloperidol and diazepam weaken the activity propagations. The data obtained for haloperidol are noteworthy, since an impaired hippocampal gating of sensory information flow from the entorhinal cortex to downstream brain structures has been implicated in the pathophysiology of schizophrenia (e.g., Javanbakht, 2006).

All slice experiments in the present study were performed in the presence of BIM (0.6 μM). We used the competitive GABA_A_ receptor antagonist BIM at this weakly blocking dose (Stepan et al., 2012) for the following reasons. First, compared to the relatively short axons of most hippocampal GABAergic interneurons (Andersen et al., 2007), projections from excitatory neurons are cut to a greater extent during preparation of slices. This probably leads to an artificial enhancement of inhibition in large-scale hippocampal circuits *in vitro* (e.g., Iijima et al., 1996). And second, the voltage-sensitive dye Di-4-ANEPPS possibly slightly potentiated GABA_A_ receptor function (Mennerick et al., 2010). Hence, BIM most likely gave rise to more physiological conditions, rather than qualitatively artificial pharmacological effects. Consistently, HTC-Waves were weakened by the GABA_A_ receptor potentiator diazepam.

We revealed that chronic social defeat stress causes markedly attenuated HTC-Waves, providing direct evidence that chronic stress can depress neuronal signal flow through the HTC. Furthermore, this finding suggests that the enhancing effects of ADs on HTC-Waves are therapeutically relevant. Importantly, fluoxetine also boosted CA3- and CA1-FDSs in slices from stressed mice and the stress paradigm used causes impaired hippocampus-dependent cognitive abilities, representing a typical symptom of depression and other stress-related psychiatric disorders (Wang et al., 2011; Wagner et al., 2012). But how could the AD effects observed contribute to an alleviation of this symptom? It is tempting to speculate that a strengthening of activity-dependent neuroplastic processes plays a critical role (Castrén, 2005). For instance, NMDA receptor-dependent long-term potentiation (LTP) at CA3-CA1 synapses (CA1 LTP), which is widely accepted to be crucial for some hippocampus-dependent cognitive abilities (Burgess et al., 2002), can be induced by HTC-Waves (Stepan et al., 2012) and possesses the properties of “cooperativity” and “associativity” (Bliss and Collingridge, 1993). Therefore, weakened activity propagations through the HTC should be less effective in the induction of CA1 LTP, whereas enhancing AD effects on them probably counteract this impairment. In this context, it is important to mention that a recent publication likewise points to an enhancement of CA1 activity by ADs. This study specifically investigated AD actions on CA1 GABAergic neurotransmission (Méndez et al., 2012). The effects observed by Méndez et al. (2012) did not depend on enhanced monoaminergic neurotransmission and required AD concentrations in the low micromolar range. Notably, investigations in humans revealed that fluoxetine and fluvoxamine reach low micromolar concentrations (10–15 μM; cf., Figure 3B) in the brain after continuous drug administration for 30 days or longer, matching the time point at which AD therapy usually starts to be effective. One or two days after start of treatment, the brain concentration is much lower (low to intermediate nanomolar range; Strauss et al., 1997; Bolo et al., 2000). It is thus conceivable that the onset of beneficial AD actions in part reflects acute drug effects on brain physiology, as it is the case for ketamine. These remarks justify the investigation of fast modulatory actions of ADs on neurophysiological processes, as done here and in many previous studies (e.g., Schofield et al., 1981; Jang et al., 2009; Méndez et al., 2012). However, further work using chronic AD administration is needed to corroborate the potential therapeutic relevance of the enhancing AD effects on HTC-Waves.

As already mentioned in the introduction section, also Airan et al. (2007) employed VSDI in brain slices to examine chronic stress and AD effects on hippocampal circuit dynamics. However, due to the partly big differences in the experimental design (see Introduction), a direct comparison with our findings is hardly achievable. Yet, if the measure “activity percolation” through area CA1 should reflect activity strength, our results do not fit to those of Airan et al. (2007). This is because our data point to an impaired neurotransmission in area CA1 of stressed mice and an opposite scenario in the presence of ADs (Figures 2G, 3C). In contrast, Airan et al. reported an increased “activity percolation” in slices from stressed rats, which is counterintuitive with regard to the well-known detrimental actions of chronic stress on the structural and functional integrity of the hippocampus, and observed an antipodal effect if animals were treated with fluoxetine or imipramine. However, one has to consider that Airan and colleagues induced CA1 activity by single-pulse stimulation of the pyramidal cell layer and the ADs were most likely washed out from their slice preparations. CA1 activity in our study resulted from burst firing of CA3 pyramidal neurons, which is a typical discharge pattern of these cells (Stepan et al., 2012). An identical comparison for the DG data cannot be drawn, since we adjusted DG activity in slices from stressed mice to predefined values.

In summary, we provide evidence for shared modulatory effects of ADs on HTC dynamics that counteract stress-induced impairment of hippocampal network function. These effects, which might be valuable for the screening for novel ADs, point to a circuit-level mechanism of ADs in the entorhinal-hippocampal system to alleviate cognitive dysfunctions often observed with depression and other stress-related psychiatric conditions. However, these effects cannot depend on enhanced neurogenesis (Hill et al., 2015). Hence, our work highlights the importance of circuit-centered approaches, complementary to investigations at the molecular/cellular level and brain imaging studies, in order to achieve a comprehensive understanding of how environmental risk factors can translate into brain disease states and how psychiatric drugs mediate their beneficial actions.

## Conflict of Interest Statement

The authors declare that the research was conducted in the absence of any commercial or financial relationships that could be construed as a potential conflict of interest.
